# Recent Advances in Bio-Based Smart Active Packaging Materials

**DOI:** 10.3390/foods11152228

**Published:** 2022-07-26

**Authors:** Tingyu Song, Sheng Qian, Tiantong Lan, Yuzhu Wu, Jingsheng Liu, Hao Zhang

**Affiliations:** National Engineering Laboratory for Wheat and Corn Deep Processing, College of Food Science and Engineering, Jilin Agricultural University, Changchun 130118, China; 13624440867@163.com (T.S.); qs15043275075@163.com (S.Q.); lttlttz@163.com (T.L.); wo_shiwuyuzhu@163.com (Y.W.); liujs1007@vip.sina.com (J.L.)

**Keywords:** bio-based materials, preparing, smart active packaging, food preservation, freshness indicating

## Abstract

The shortage of oil resources is currently a global problem. The use of renewable resources instead of non-renewable ones has become a hot topic of research in the eyes of scientists. In the food industry, there is a lot of interest in bio-based smart active packaging that meets the concept of sustainability and ensures safety. The packaging has antibacterial and antioxidant properties that extend the shelf life of food. Its ability to monitor the freshness of food in real time is also beneficial to consumers’ judgement of food safety. This paper summarises the main raw materials for the preparation of bio-based smart active packaging, including proteins, polysaccharides and composite materials. The current status of the preparation method of bio-based smart active packaging and its application in food preservation is summarised. The future development trend in the field of food packaging is foreseen, so as to provide a reference for the improvement of bio-based smart active packaging materials.

## 1. Introduction

Food packaging materials are an important part of the food supply. As a key to ensuring the quality of food, people are becoming more and more careful in their choice of packaging materials. Plastics have been widely used in the packaging of various products and have been developed for their low cost and good mechanical properties. However, plastics are mostly petroleum-based polymers, which are difficult to degrade. A large proportion of plastic waste is sent to incineration plants or landfills [1], where large amounts of carbon dioxide and other toxic compounds are produced during the disposal process, thus contributing to global warming [2]. If the harmful substances return to the food chain, it will have a long-term negative impact on human beings [3,4]. In the current environment of green and low-carbon living, the development of nonbiodegradable plastic products has created limitations, and the resulting waste of resources and environmental pollution is contrary to the concept of sustainable development [5]. In order to meet the development of the times, the use of renewable resources instead of non-renewable resources has become a hot spot for research. Polymers are commonly used in food packaging and are favoured by researchers for their better oxygen barrier, moisture resistance and crosslinking properties [6]. Compared to nonbiodegradable polymers, biodegradable natural polymers are more in line with the requirements of sustainable development, making them a better choice for the preparation of food packaging materials.

However, while people are concerned about the choice of packaging materials, they should also pay more attention to the safety of the packaging. Food safety is closely related to human life and health. The main role of food packaging is to protect food from external environmental contamination, which can prevent food aromas, flavours and colours from migrating into the environment. For some special foods, packaging materials can also be prepared. Hydrophilic packaging can prevent the absorption of oils and fats and is suitable for covering diet foods [7]. Microbial contamination caused by exposure to the environment during the packaging or processing of food is a serious problem [8]. Traditional methods such as heating, freezing or drying are effective but still inadequate in inhibiting microbial growth. Some researchers have incorporated antimicrobial agents into polymer films to obtain antimicrobial packaging that can inhibit the growth of microorganisms and also ensure food safety [9,10]. The existence of antimicrobial packaging largely extends the shelf-life of food products, but it does not prevent food spoilage, which can occur at any time and leaves consumers unable to judge the quality of the food. To solve these problems, smart packaging that can sense and monitor changes inside food and transmit information to the outside world has emerged [11]. By allowing consumers to visualise changes in the state of the packaging material, they are able to avoid wasting food and resources to a certain extent. Despite the development of alternative food packaging materials to plastic, the issue of meeting all sustainability criteria and ensuring that the packaging remains in good condition during transport remains to be addressed [12]. In this paper, we review the types of bio-based smart active packaging materials. Protein-based materials include whey protein, collagen, zein and soy protein isolate, while polysaccharide-based materials include starch, chitosan, sodium alginate and pullulan. We also present methods for the preparation of smart active packaging, including the casting method, electrostatic spinning technology and 3D printing technology, and compare the advantages and disadvantages of the three techniques, as well as their scope of application. Based on the current research status, the development trend of bio-based smart active packaging is discussed to provide a basis for the future development of food packaging materials.

## 2. Types of Bio-Based Materials

Biodegradable materials are divided into naturally degradable materials and chemically synthetic degradable materials. Naturally degradable materials include proteins, polysaccharides and lipids, which are abundant and guaranteed safe. Proteins and polysaccharides are the main materials used to make active packaging [13]. The functionality of protein-based packaging materials depends mainly on the extension of protein chains and amino acid sequences. Protein denaturation is achieved by means of acids, bases and heat to form the additional extension system needed for packaging materials, and the denatured proteins will be more cohesive. The ordered hydrogen bonding network of polysaccharides facilitates tight bonding between adjacent chains and ensures less deterioration of food products due to oxidation. Compared to proteins and polysaccharides, lipids are more difficult in forming cohesive and self-contained packaging materials, and therefore, less research has been carried out to design packaging materials from lipids. Compared to naturally biodegradable materials, chemically synthesised materials are too costly to use in food packaging due to their complex and expensive processing; therefore, the following section focuses on the application of naturally biodegradable materials in food packaging. Table 1 summarises the advantages and disadvantages that exist for each type of material [13].

### 2.1. Protein-Based Materials

#### 2.1.1. Whey Protein

Whey protein is an animal protein with high nutritional value, containing the eight essential amino acids in a ratio close to the human body’s needs, and is easily digested and absorbed. Among vegetable proteins, only soya protein is a complete protein, but soya protein is not as good as high-quality animal protein in terms of absorption. Whey protein contains 50–55% beta-lactoglobulin, 20–25% alpha-lactalbumin, 10–15% immunoglobulin, 1 to 2% lactoferrin and a number of active ingredients, which make it an important substance for human growth and development. This makes whey protein an important substance for human growth and development.

The properties of whey protein are very popular among researchers, and attempts have been made to develop its application in more areas. In research, it has been found that whey protein is safe and nontoxic, rich in a variety of bioactive components and can form good performance films and biodegradable coatings for industrial applications [14]. Its characteristics are in line with the general trend of saving resources and protecting the environment in today’s society, and it is a good biological material that can replace non-renewable resources. Films made from whey proteins are viscous and stretchable and show a high barrier to oxygen and aromas due to their tightly packed, ordered network structure and low solubility [15]. However, whey protein has a high hydrophilic amino acid content, and the mechanical strength and vapour barrier properties of products made from pure whey protein materials may be poor [16], so the addition of plasticisers, antioxidants or antimicrobial agents may be considered to improve their mechanical strength and enhance their functional properties. The high cost of whey proteins due to their high price and their extensive use in industrial production has been a drawback to their development, but because of their inherent high-quality functional properties, the blending of whey proteins as active ingredients with other materials can be used to achieve enhanced functional properties. Paolo Pino et al. [17] made ZnO nanocomposite films using whey protein as a matrix and characterised their physical and functional properties to test their antibacterial activity. The results showed that zinc oxide was successfully included in the protein matrix, and the FTIR, XDR and swelling tests showed that there was only a weak physical interaction between the two species of the composite, with significant inhibition of the Staphylococcus epidermis used in the tests. The whey protein–zinc oxide nanocomposites were shown to be beneficial as packaging materials for use in the antimicrobial preservation of food products.

#### 2.1.2. Collagen

Collagen is one of the main components of the extracellular matrix of animal connective tissue and is the most abundant and widely distributed functional protein in mammals [18,19]. Proline and hydroxyproline are amino acids that are unique to collagen, and the number of hydrogen bonds they form plays an important role in the stability of the collagen structure. In the food industry, collagen is used both in its native form as a food additive and in the form of collagen biomaterials [18].

In addition to mammalian collagen, marine collagen can also be extracted from the tissues of marine organisms. Compared to mammalian collagen, marine collagen presents a comparable or slightly lower molecular weight and a lower denaturation (melting) temperature. To improve the thermal stability, marine collagen can be combined with suitable crosslinking treatments. The preparation of collagen films is usually achieved by using a plasticiser, mainly glycerol in the range 20–30 wt%, a small molecule of low volatility added to decrease attractive intermolecular forces along polymer chains and increase the free volume and chain mobility [20]. The ability of collagen membranes to shrink and stretch allows them to be used as edible enteric coatings in meat processing [21]. However, the poor thermal stability, poor mechanical properties and hydrophilicity of collagen membranes have limited their development. Many efforts have been made by researchers to overcome these difficulties.

In tests, researchers have demonstrated that the addition of antibacterial tea polyphenols to collagen casings significantly improves the mechanical properties of the casings and increases the antibacterial activity against Escherichia coli, Staphylococcus aureus, Bacillus subtilis and Salmonella [22]. Collagen can also be modified by crosslinking with other proteins that are thermally stable [23]. The test results show that the thermal stability, tensile strength, elongation strength at break and FTIR of the complexes have been measured and demonstrate that the thermal stability, tensile strength and elongation at break of the complexes obtained by crosslinking collagen with heat-resistant proteins have been improved.

#### 2.1.3. Zein

Zein is the main storage protein in maize endosperm and is obtained from maize by-products [24]. Its content ranks first among maize proteins, accounting for approximately 60%, and is a renewable, biodegradable biopolymer that is widely used in drug delivery, food packaging, cosmetics, textiles and other applications [25,26]. Zein has exceptional solubility due to its high content of hydrophobic amino acids such as proline, leucine and alanine [27]; insolubility in water but solubility in aqueous ethanol solutions and highly concentrated alkaline solutions and film-forming properties, all of which depend on its special amino acid structure. Zein is widely available, safe and nontoxic; in addition to having good film-forming properties, it also has good gas barrier properties. In the storage and transportation of fresh fruits and vegetables, the functional properties of zein enable it to be used as a coating material to preserve fresh fruits and vegetables and has a broad development prospect in the field of food packaging.

However, many studies have shown that the mechanical properties and stability of zein films made from a single component are poor, and their application is limited, so it is extremely important to study the modifications of proteins, such as adding plasticisers and crosslinkers to the preparation of zein films to improve their performance. In the current research on the use of the maize alcohol-soluble protein as a food packaging material for fish, meat and other foods with high lipid contents for packaging, it has the effect of delaying the oxidation of food and inhibiting the growth of microorganisms; for fresh fruits and vegetables for packaging, it has the effect of preventing browning and extending the shelf life of fruits and vegetables. Liming Zhang et al. [28] prepared a unidirectional water barrier film with a moisture proofing outer layer and hydration inner layer using zein and chitosan as raw materials using a layer-by-layer casting method. The TGA showed that the addition of zein improved the thermal stability of the films. The layer-by-layer assembly coating improved the elongation and tensile strength of the films and reduced the water vapour and oxygen transmission rate. Debao Wang et al. [29] prepared antimicrobial nanofiber packaging films by loading perillaldehyde into gelatine/zein polymers. The water contact angle results showed that the hydrophilicity of the fibrous films gradually changed to hydrophobicity with the increase of perillaldehyde. The thermal analysis showed that the gelatine/zein/perillaldehyde (5:1:0.02) films had good thermal stability.

It can be seen that there is great scope for the development of zein in the field of food packaging, and as a renewable resource, the trend is to gradually reduce the use of non-renewable resources, and to increase the development and use of renewable resources will become the research goal of more researchers.

#### 2.1.4. Soy Protein Isolate

Soy protein is rich in protein, with a mass fraction between 40% and 50%, and has a high digestibility and absorption rate compared to the other vegetable proteins. Soy protein is a very high-quality vegetable protein resource, as it is more in line with the composition of essential amino acids and has sufficient contents. The strong intermolecular crosslinking of soya bean proteins gives it good film-forming properties, and the biocompatibility and biodegradability of soya bean proteins are conducive to their development in the food packaging sector [30].

However, the presence of a large number of hydrophobic amino acid residues in soy protein isolate can make soy protein isolate form a spherical structure with the hydrophobic centre inside and the hydrophilic outer layer outside, which, in turn, leads to the poor water resistance of the soy protein isolate membrane [31]. In order to expand the range of applications for the soy protein isolate, physical methods such as heating, ultrasound and ultra-high pressure can be used to alter the molecular structure of the protein and change its properties. Researchers at home and abroad have found that the addition of nanoparticles to soy protein isolate membranes can improve the strength, toughness and water barrier properties of the membranes [32].

Ran Tao et al. [33] added different concentrations of carvacrol and glycerol in the preparation of soy protein isolate films. The effects of carvacrol and glycerol on the film properties were investigated by examining the mechanical properties, opacity, water vapour permeability and antibacterial properties. The results showed that carvacrol and glycerol improved the ductility of the soy protein isolate films and acted as plasticisers. The concentration of glycerol was proportional to the water vapour permeability of the films, and the higher the concentration of glycerol, the higher the water vapour permeability of the films.

### 2.2. Polysaccharide-Based Materials

#### 2.2.1. Starch

Starch is a polysaccharide made up of glucose molecules, which are abundant and inexpensive and are often found in nature in the form of granules. Due to its good film-forming and biocompatibility properties and its hydroxyl structure, starch can be easily regulated by chemical or biological enzymes and is therefore considered an ideal natural biodegradable material. Some researchers are using starch as a packaging substrate in the food packaging sector. When a starch solution is continuously heated, the starch granules swell with water until they rupture, forming a colloidal solution. This is the process of pasting, which is essentially caused by the breaking of hydrogen bonds that maintain the stability in starch under conditions of water and heat. Under low-temperature conditions, the starch molecules are reformed into an ordered structure by hydrogen bonding, which is starch regrowth. When preparing starch-based biodegradable packaging materials, the retrogradation of starch can lead to poor mechanical properties of the prepared packaging material. Moreover, the high hydrophilicity of starch can limit its use in practical production [34]. In order to improve the functional properties of starch-based packaging materials, additives such as crosslinkers and plasticisers are usually added during the preparation process to make them meet the requirements of food packaging materials.

Ain Nadirah Romainor et al. [35] investigated the effect of the composition of starch, citric acid and polyvinyl alcohol on the preparation of starch–citrate films and their antimicrobial effect. The natural starch itself did not possess antimicrobial activity, but the starch–citrate films made with the addition of antimicrobial agents could suppress 98 to 99% of foodborne bacteria growth and 87–99% of fungal growth. This shows that starch–citrate films have good prospects in the field of antimicrobial packaging.

#### 2.2.2. Chitosan

Chitosan is synthesised by the deacetylation of chitin, the only positively charged alkaline polysaccharide in nature [36,37,38]. It is nontoxic and odourless and can undergo chemical reactions such as oxidation, reduction, nitration, carboxymethylation and hydrolysis under the corresponding conditions to produce various chitosan derivatives with different properties. The positively charged nature of chitosan allows it to establish electrostatic interactions with other compounds [39]. Polyphenolic compounds may interact with the hydroxyl or amino groups on the chitosan molecular chain by intermolecular hydrogen bonding or electrostatic interactions, thus changing the functional properties of chitosan.

As a functional oligosaccharide, it has good film-forming properties, biocompatibility and biodegradability, enabling it to be used in industrial production as a biodegradable food packaging material. Chitosan is widely used in the preparation of water-blocking films, and to improve its stability, it is often blended with polymers with more functional chains, such as polyvinyl alcohol [40]. Polyphenolic compounds may interact with the hydroxyl or amino groups on the chitosan chain in intermolecular hydrogen bonds or electrostatic interactions, thus altering the functional properties of chitosan. Victor Gomes Lauriano Souza et al. [41] used chitosan and montmorillonite as materials to which ginger essential oil was added to produce bio-nanocomposite films and characterised their physical and functional properties. The chitosan itself has good oxygen barrier properties, which are enhanced by the addition of montmorillonite, and can more effectively delay the oxidation process of food products. The nanomaterials also increased their UV-blocking ability, which will be better developed in food preservation.

#### 2.2.3. Sodium Alginate

Seaweed is a fast-growing and widely sourced organism, which contains a large number of polysaccharides, proteins, minerals, vitamins and other bioactive compounds. Seaweed polysaccharides have become a research hotspot due to their film-forming, biodegradable, antioxidant and antibacterial properties [42]. Sodium alginate is a type of algal polysaccharide, which is a linear polymer consisting of β-D-mannuronic acid (M) and α-L-gulo-glucuronic acid (G) linked together by a 1-4 glycosidic bond. It is a preferred material for making green packaging for food due to its long polymer chain, safety, nontoxicity and easy availability [43]. Hao Cheng et al. [44] prepared a sodium alginate-sophora bean gum active film containing ryanodine and measured its physical, antioxidant and antibacterial properties. The results showed that the appropriate amount of daphnetin could improve the optical, mechanical and barrier properties of the active film, and the antioxidant and antibacterial properties of the active film were enhanced with the increase of the daphnetin content. Haifa Mohammed Alghamdi et al. [45] prepared NaAlg/PEO/Ni/ZnO nanoparticle polymer nanocomposite samples using the solution casting method and applied them to bioactive food packaging. After a series of characterisations of the films, it was demonstrated that the incorporation of nanoparticles improved the properties of the nanocomposites. The tensile strength, stiffness and Young’s Modulus of the nanocomposites were all improved. In conducting antimicrobial tests, it was found that the sodium alginate films with the addition of nanoparticles showed enhanced activity against Escherichia coli, Staphylococcus aureus, fungus and yeast.

#### 2.2.4. Pullulan

Pullulan is a microbial polysaccharide, a linear polymer consisting of maltotriose units linked by alpha-1,6 glycosidic bonds [46]. It is readily soluble in water, nontoxic and non-hazardous and is biodegradable in nature and is therefore widely used in food, pharmaceutical and chemical applications. Pullulan also has good oxygen barrier and film-forming properties, making it promising for functional biomolecules. Some studies have confirmed that pullulan as a film-coating preservative has a significant effect in extending the shelf life of products such as fruits and vegetables, but the fragility and high production cost of pure pullulan films limit their wider applicability [47]. To improve these deficiencies, researchers have combined pullulan with other polymers to make composite membranes or combined bioactive substances with pullulan to slowly release the active compounds into the food, which can improve the properties of the membranes. The antimicrobial properties of pullulan also help to inhibit microbial growth, reduce food spoilage and reduce nutrient loss [48]. Nuno H.C.S. Silva et al. [49] prepared nanocomposite films from pullulan and lysozyme nanofibers using the solution casting method. The mechanical properties, thermal stability, antioxidant activity and antimicrobial properties were tested, showing that the nanocomposites produced had good mechanical properties and thermal stability up to 225 °C and antioxidant activity of about 77%. The results of the antibacterial activity test showed that the antibacterial effect of the composite film increased with the increase of the nanofiber content.

### 2.3. Composite Materials

Although each of the above-mentioned active packaging materials has its own advantages, there are various disadvantages in their practical applications. A single base material may not be sufficient to solve the problems encountered during actual production, so some researchers are exploring composite packaging materials, which combine polysaccharides, proteins, lipids and other polymeric substances, to complement each other and add certain active substances to them, so as to improve the functional properties of packaging materials. Common composite materials include protein–polysaccharide materials, protein–protein materials and polysaccharide–polysaccharide materials. Mehrdad Khanzadi et al. [50] prepared and characterised whey protein–pullulan composite membranes using glycerol as a plasticiser to derive the best-performing membrane by adjusting the ratio of glycerol. It was found that the presence of lipids helped to reduce the water vapour transport, enhance the strength and ensure the structural integrity of the protein–polysaccharide composite membranes due to the presence of lipids. Jorge Iván Castro et al. [51] studied and characterised the biofilms obtained from mixtures of tapioca starch and gelatine, showing that the films prepared with glycerol as a plasticiser and a 53/47 ratio of tapioca starch to gelatine had the best overall evaluation, and their mechanical properties were close to those of low-density polyethylene materials and could be used as food packaging materials.

## 3. Methods for Preparing Reactive Packaging Materials

Bio-based active packaging is characterised by the addition of antibacterial agents, antioxidants, absorbents and releasers to the packaging material to regulate the environment inside the packaging to ensure freshness and extend the shelf life of the food. In the process of making packaging materials mainly used in the flow casting method, coating method, extrusion blow moulding method, etc. [52], with the continuous development of technology, electrostatic spinning technology and 3D printing technology have also gradually been applied to the preparation of packaging materials [53]. The advantages and disadvantages of the three preparation methods are summarised in Table 2.

### 3.1. Casting Method

**Figure 1:**
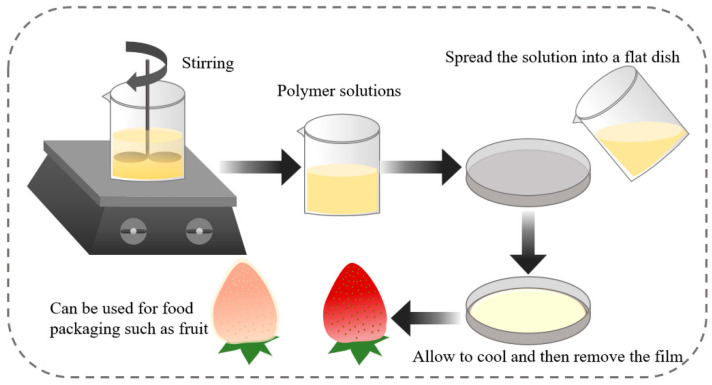
Schematic diagram of the preparation of packaging materials by the flow casting method.

In most of the studies, the solution flow method is used, whereby the mixed solution is spread directly into a Petri dish, dried with a blower and then uncoated after cooling. This method of production is fast, productive and easy to use and is commonly used in the packaging of foodstuffs, daily necessities, etc. This method is commonly used in the preparation of polypropylene films and can also be laminated with PET and BOPP films to produce composite films. It is also used in the preparation of bio-based packaging materials. Figure 1 shows a schematic diagram of the preparation of packaging materials by the flow casting method.

### 3.2. Electrostatic Spinning Technology

**Figure 2:**
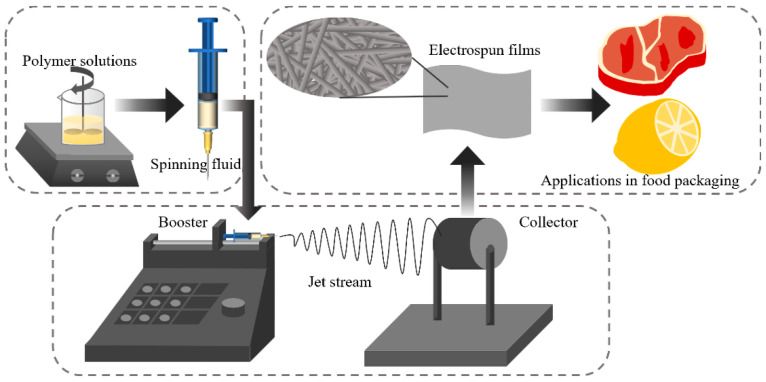
Schematic diagram of the electrospinning equipment.

Electrostatic spinning is a nonthermal processing technology where a positively charged polymer solution is pumped through a syringe drop by drop at a high voltage; the electric field causes the droplets to form a Taylor cone, due to the presence of elongational viscosity, thus forming a continuous and stable jet [54], Figure 2 shows a schematic diagram of the electrostatic spinning equipment. Compared to the traditional thermal processing methods, electrostatic spinning technology can effectively protect thermally unstable active substances, has less impact on bioactive substances and does not require strict environmental conditions such as temperature, pressure or chemical substances during the preparation process, making it one of the simplest and most effective methods for preparing nanofibers at this stage [55]. The electrostatic spinning technique allows the polymer to form nanofibers with a larger specific surface area when preparing nanocomplexes. The ability of natural polymers to load active substances can enable them to release the encapsulated compounds over a certain period of time, which, in practice, can effectively prevent the oxidation of the encapsulated food and avoid contamination by microorganisms [56]. It has been found that konjac glucomannan is a safe and edible polysaccharide but has some drawbacks in its application as a food active packaging film due to its strong hydrophilicity and lack of mechanical properties and gas barrier. To improve the physical properties of konjac glucomannan, some researchers have used electrostatic spinning technology instead of the traditional solution casting method. The results show that the biocompatibility, antioxidant and antibacterial properties of the fibrous films prepared by the electrostatic spinning method have been improved [57].

### 3.3. Three-Dimensional Printing Technology

**Figure 3:**
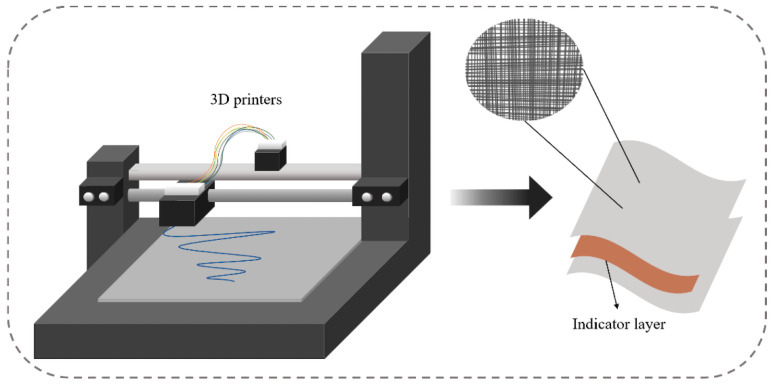
Diagram of a 3D printer.

Three-dimensional printing is a technology for manufacturing objects based on 3D mathematical model data, forming the target object quickly and accurately by continuous physical stacking [58]. Compared to traditional processes, 3D printing allows for more complex products to be designed according to consumer needs and reduces material waste. Common 3D printing methods include stereolithography and extrusion-based 3D printing. In the food packaging sector, extrusion-based 3D printing is often used to fabricate different types of sensors, indicators and tag prototypes [53]. Extrusion-based 3D printing is also known as fused deposition modelling. By extruding a filamentary material, such as thermoplastic, from a heated nozzle, it is deposited on a predetermined trajectory. With each completed layer, the table is lowered to deposit a new layer in a stack. Figure 3 shows a schematic diagram of a model based on extrusion-based 3D printing technology.

## 4. Bio-Based Smart Active Packaging Materials for Food Applications

### 4.1. Application in Food Preservation

In the preparation of bioactive packaging, the base materials are mainly divided into protein-based materials, polysaccharide-based materials and composite materials. Compared to food packaging materials made from a single base material, composite packaging materials will have stronger mechanical properties, water resistance and oxygen barriers. The method of compound modification has been a hot research topic in recent years, where two or more polymeric substances are compounded with each other, enabling their advantages to complement each other and facilitating their maximum effect.

**Figure 4:**
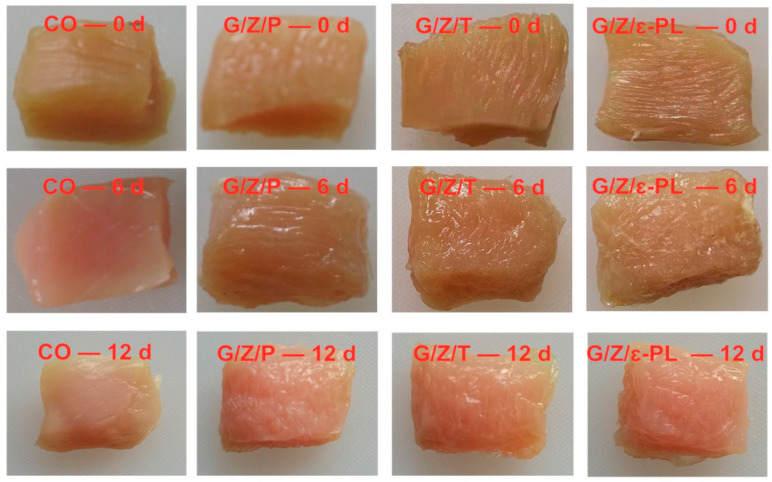
The images of the preservation effect of the gelatine/zein/perillaldehyde, gelatine/zein/thymol and gelatine/zein/ε-polylysine nanofiber films on chicken breasts (taken from [60]).

Zengliu Song et al. [59] prepared collagen/zein electrospun films incorporated with gallic acid with the electrospinning technique and used it for the preservation of tilapia muscle. After the characterisation of the microstructure and physical properties of the electrospun membrane, it was demonstrated that gallic acid was uniformly dispersed in the collagen/zein electrospun films, and the three substances were bound to each other through intermolecular interactions. The addition of gallic acid enhanced the antioxidant properties of the electrospun membrane and had a significant effect on the preservation of tilapia muscle. Debao Wang et al. [60] made and characterised three edible bioactive packaging films by loading perillaldehyde, thymol and ε-polylysine in gelatine–zein nanofibers and evaluated the functional properties of the composite films by their effect on the preservation of frozen chicken breast meat. The results showed that the elongation, elongation and thermal stability of the laminates with the addition of perillaldehyde were improved, and the water and oxygen permeability were lower than those of the laminates with the other two actives. Furthermore, the frozen chicken breasts packed with the films containing perillaldehyde had the lowest total viable count during storage, effectively extending the shelf life of the food, as Figure 4 shows.

Active food packaging materials are mainly biopolymers with the addition of natural active substances [61], but care needs to be taken when selecting the active substances. While the addition of some active substances may have antibacterial and antioxidant effects, the possible presence of irritating odours from the active substances can reduce the organoleptic quality of the product. Natural polyphenolic compounds such as curcumin, anthocyanins, gallic acid and catechins are currently used in the preparation of biopolymer-based active packaging films [62].

Maria Tsironi et al. [63] investigated the effect of natural antimicrobial agents on enhancing the properties of whey protein by preparing whey protein films supplemented with ginger and rosemary essential oils and wrapping lamb with them to determine the inhibition of microorganisms by the films. The results showed that whey protein films supplemented with 1% ginger and 1% rosemary essential oils had a significant inhibition effect on lamb, and the TBARS values remained low, indicating a significant delay in the oxidation of the meat samples. Vasiliki G. Kontogianni et al. [64] used whey protein concentrate and aqueous extracts of rosemary and sage to prepare edible active packaging materials. The films were characterised according to their physicochemical and tensile properties and tested for their antioxidant activity. The results showed that the films incorporating the sage infusion had enhanced mechanical properties. The material was used to package cheese, which showed a good preservation of freshness.

### 4.2. Application in Indicating Food Freshness

The presence of active packaging plays a vital role in food preservation, as its antibacterial and antioxidant effects can not only extend the shelf life of food but also effectively prevent the waste of resources and play a key role in delaying food spoilage. However, spoilage is inevitable during the storage of food, for example, food spoilage of animal origin is closely linked to microorganisms such as Pseudomonas, Bacillus, Escherichia coli and Salmonella, which not only affects the quality of the food but also endangers human life and health. Chemical sensors, fluorescent sensors and electronic noses are too time-consuming to determine whether food is spoiled [65], so there is an urgent need to develop technologies that can visually determine the freshness of food. In response to this phenomenon, some researchers have developed smart active packaging with indicators that can monitor the freshness of food in real time.

Intelligent and active packaging is the preparation of packaging materials in which environmentally sensitive materials with indicators are added, indicators of time to sense changes in the environment and through the appearance of the packaging material to convey the information. In the storage process, fruits and vegetables will produce ethylene and CO_2_, meat products in the process of storage under the action of microorganisms and enzymes will release hydrogen sulphide gas, the content of total volatile salt nitrogen [66] and fish products will be left for a period of time, and the content of nitrogen compounds will rise; according to these characteristics of the research, to choose the appropriate indicators for different packaging food, there are microbial-sensitive indicators or gas-sensitive indicators. These indicators are microbiologically sensitive or gas-sensitive. Most smart packaging materials are based on pH-responsive colorimetric indicators that change by visual colour, such as time–temperature indicators, freshness indicators and leak indicators [67]. The natural pigment anthocyanin is one of the most popular dyes, containing highly phenolic compounds with a wide range of pH responses [68,69]. The outstanding active role of natural pigment anthocyanin allows it to make some contributions in food preservation as well [70].

**Figure 5:**
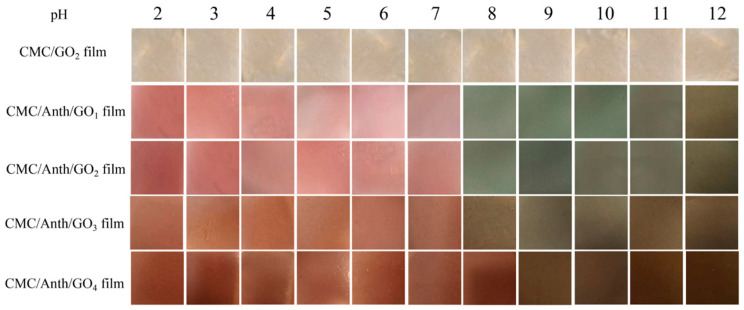
The colour response of films to different pH buffer solutions after soaking for 1 min (taken from [73]).

Zahra Aghaei et al. [71] designed a protein discolouration nanosensor to assess the quality of salmon with electrostatically spinning nanofibers by using zein as the substrate and alizarin as the indicator. The sensor changed colour over time from yellow to magenta, and the microbial content of the salmon proved that the salmon had deteriorated at the time of the colour change. Ruichang Gao et al. [72] prepared a colorimetric indicator film for monitoring the freshness of milk. The presence of gelatine facilitated the interaction between the components, as observed by scanning electron microscopy, using zein as a matrix into which blueberry anthocyanins, gelatine and Fe^2+^ were compounded by using the electrostatic spinning method. The colour change of the indicator film with the addition of gelatine and Fe^2+^ was more pronounced than that of the control indicator film in solutions of different pH values (3–7) and in milk stored for different times. Yali Zhao et al. [73] used carboxymethyl cellulose as a substrate, anthocyanin as an indicator and graphene oxide as an enhancer to prepare pH-sensitive smart reactive packaging films and used them to package fresh pork to confirm the sensitivity of the reactive films to pH. By adjusting the content of graphene, the water barrier, tensile strength and gas barrier of the film were improved, and the colour of the packaging changed significantly under different pH conditions, alerting consumers in real time when food deterioration occurred, as Figure 5 shows.

## 5. Conclusions

In summary, the good water barrier, gas barrier, tensile strength and denseness of bio-based smart active packaging materials play crucial roles in the preservation of foodstuffs. Active substances such as crosslinkers, plasticisers and antimicrobial agents will improve the functional properties of packaging materials in different ways, and smart active packaging materials prepared by adding indicators will help in the monitoring of food safety. Despite the increasingly systematic research on bio-intelligent active packaging materials, there are still some pressing issues to be resolved in practical applications. In the future of bio-smart active packaging, the issues that need improvement focus on the following areas:

① The raw materials used in the preparation of smart packaging are both naturally derived and synthetic. It is difficult to guarantee the safety of synthetic substances, and therefore, there is a lack of toxicological testing on synthetic colourants. Smart packaging materials exist to improve food safety, and a poor choice of raw materials can lead to counterproductive test results.
② The choice of indicator also determines the effectiveness of the application of the product. The majority of studies have been carried out using pH changes to change the colour of the indicator, but in practice, environmental conditions can easily affect the colour development of the indicator, and changes in the external factors can have an impact on the accuracy of the indicator. Moreover, the colour development effect of some indicators is not obvious, and it is difficult to judge subtle colour changes by the naked eye, and even the colour change of some indicators can be reversed, which further increases the impact on the accuracy of smart packaging.
③ Further research is needed into the kinetics of active substance diffusion. The rate of diffusion of the active substance affects the quality of the food, and a model for the slow release of the active substance needs to be developed.
④ The technology used in the preparation of packaging materials still needs to be improved. Due to the diversity of the preparation methods, there are also gaps in the performances of the packaging materials they produce. In an analysis of the current state of research at home and abroad, it was found that the performances of packaging materials largely determine their applications and that poor mechanical properties due to imperfect preparation techniques can seriously undermine the functionality of a product.

## Figures and Tables

**Table 1:** Advantages and disadvantages of various types of packaging materials.

CATEGORY		ADVANTAGES	DISADVANTAGES
**PROTEIN**	Whey protein	The protein has been deformed by acids, alkaline solvents and heat to create the additional extension system required for proteins with a more cohesive structure.	Packaging materials prepared from a single protein have poor mechanical strength and resistance to water and become brittle after drying.
	Collagen		
	Zein		
	Soy protein isolate		
**POLYSACCHARIDES**	Starch	The ordered hydrogen bonding network of polysaccharides facilitates close bonding between adjacent chains and good oxygen barrier properties.	The presence of the hydrophilic structural domain of the polysaccharide leads to its poor water barrier properties.
	Chitosan		
	Sodium alginate		
	Pullulan		
**COMPOSITE MATERIALS**		The use of two or more polymers and a number of actives allows better control of release properties and enhances the functionality of the packaging material.	The preparation process is more complex than for a single material, and it is important to ensure that the substances added act synergistically rather than antagonistically.

**Table 2:** Advantages and disadvantages of the three preparation methods.

PREPARATION METHODS	ADVANTAGES	DISADVANTAGES
**CASTING METHOD**	simple, fast and cost-effective operation	uneven thickness of the product, poor tensile strength and modulus of elasticity of the product
**ELECTROSTATIC SPINNING TECHNOLOGY**	fast, low cost, high specific surface area, high porosity and homogeneous fibres	the equipment parameters are not easily adjustable, and the preparation process is prone to failure
**3D PRINTING TECHNOLOGY**	complex structures in multiple materials can be printed simultaneously using multiple nozzles at low cost	time-consuming preparation process and more complex than other preparation methods

## Data Availability

Data is contained within the article.
